# Super-critical fluid extract of *Bryonopsis laciniosa *(*Shivlingi*) seeds restores fertility in zebrafish models through revival of cytological and anatomical features

**DOI:** 10.1186/s13048-022-00982-6

**Published:** 2022-04-27

**Authors:** Acharya Balkrishna, Pradeep Nain, Monali Joshi, Brijesh Kumar, Anurag Varshney

**Affiliations:** 1 Drug Discovery and Development Division, Patanjali Research Institute, NH-58, Haridwar, 249 405 Uttarakhand India; 2 Department of Allied and Applied Sciences, University of Patanjali, Patanjali Yog Peeth, Roorkee-Haridwar Road, Haridwar, 249 405 Uttarakhand India; 3 Special Centre for Systems Medicine, Jawaharlal Nehru University, New Delhi, India

**Keywords:** *Bryonopsis laciniosa*, *Shivlingi* seed oil, N-ethyl-N-nitrosourea mutagenesis, Impaired fertility, Zebrafish, β-sitosterol, Stigmasterol

## Abstract

**Background:**

The Ayurvedic system of medicine mentions the use of seeds of *Bryonopsis laciniosa* (L.) Naud. (also known as *Shivlingi* due to their unique structure resembling a ‘*Shivling’*) for treating sexual dysfunction, impaired fertility, and as a general virility-booster in both males and females. To investigate the scientific basis for such claims, the current study was designed for the chemical characterization of the super critical fluid extracted *Shivlingi* seed oil (SLSO), and subsequent evaluation of its reproductive fecundity in the zebrafish model of N-ethyl-N-nitrosourea induced infertility.

**Results:**

Linoleic and linolenic acids were the major fatty acids in the SLSO, with trace amounts of β-sitosterol and stigmasterol. Both male and female zebrafish, when fed orally with the SLSO infused pallets, showed a dose-dependent increase in fertility and fecundity rates. Microscopic observations revealed recovery in the gross ovarian anatomy and consequential improvement in egg production in infertile female zebrafish. Similarly, cytological studies exhibited increased sperm counts and motility in male zebrafish. SLSO exhibited effects similar to the human equivalent dose of Letrozole.

**Conclusion:**

Taken together, these observations demonstrated the fertility-boosting potentials of SLSO comparable to the widely used infertility drugs. As a whole, this research work has provided scientific evidence for the rationale behind the use of *Shivlingi* seeds in Ayurvedic treatment for infertility in humans. Finally, but importantly, this study warrants further scientific investigations into different aspects of SLSO on human reproductive health.

## Background

Fertility and problems associated with it are among one of the most complex issues in medical science. The World Health Organization (WHO) has classified infertility as a disability and a ‘disease of the reproductive system’. Infertility is estimated to affect approximately 186 million people worldwide, with 10% of women and 12% of men [1, 2]. Increase in cases of infertility and subfertility has been attributed to modern lifestyles. As a result, there is a great demand for treatments for fertility-related issues. Although development of in vitro fertilization (IVF) has helped in decreasing the infertility rate, this artificial method of conception may not be easily acceptable among a wider population. The major causes of female infertility include an age-dependent decrease in the number and quality of oocytes, physical abnormalities in the female reproductive tract, and diseases that affect ovarian health, all of which can lead to developmental abnormalities and spontaneous loss of embryo [3, 4]. Among males, the major causes of infertility include sexual dysfunction, and a decrease in the quality and quantity of sperm. Hypogonadism leading to decreased testosterone levels can also affect spermatogenesis [5, 6]. One of the most commonly used treatments for reducing female infertility is the induction of ovulation through aromatase inhibitors, which are believed to reduce the level of estrogen by blocking the aromatase enzyme, and concomitant conversion of androgens to estrogen in the ovary [5]. Its use is reported to increase the follicular number and endometrial health [6]. Similarly, Letrozole and Clomiphene are also used widely to treat fertility in females and males, respectively. However, reports have highlighted increased cases of ovarian hyper-stimulation syndrome leading to multiple gestations and increased preterm births. Moreover, neonatal morbidities including gestational diabetes and locomotor malformation have also been reported upon treatment with letrozole and gonadotropins [7]. In light of such serious morbidities associated with infertility treatments, and increased awareness of traditional and herbal medicines, there is an increase in the demand for safe alternative natural treatments with minimal side-effects [8].

The Ayurvedic system of medicine advocates the use of *Bryonopsis laciniosa* (*Shivlingi*) seeds (Cucurbitaceae family) for the treatment of infertility since ancient times [9, 10]. Being a herbal product, it has extremely low toxicity with minimal side-effects on the human body. Apart from being used as a medicine, it can be taken as a dietary supplement to improve the health and vitality of the human body. [11] Numerous Ayurvedic references establish the use of these seeds to restore fertility in both men and women, such as *Rāja Nighaṇṭu* (*Guḍūcyādi-Varga*) and *Śāligrāma-nighaṇṭu* (*Guḍūcyādi-Varga*) from the 14 and nineteenth centuries, respectively. It is an annual climber with bright red fruits and is used as a hypoglycemic herb in some parts of India. However, its most recognized use, as orally consumed whole seeds, is in the improvement of sexual behaviour and treating infertility [12, 13]. However, despite an established medicinal use in Ayurveda, neither the phytochemical constitution of these seeds nor their probable mode-of-action as a fertility enhancer is known.

Traditional systems of medicine used crude preparations of *Shivlingi* seeds for the treatment of fertility problems [14]. With advancements in technology, such as the Super Critical Fluid Extraction (SCFE) process, it is now possible to extract oil from different sources in a reproducible and environment-friendly manner. The use of gases in liquid form under high pressure as Super Critical Fluids, instead of organic solvents, allows the extraction of non-polar phyto-constituents without any unwanted residues, which might require safe disposal methods [15, 16]. Food grade liquid carbon dioxide (SC-CO_2_) promotes high-quality extraction with minimal thermal degradation compared to traditional extraction methods. This method of extraction also allows changing the process parameters for optimum recovery of specific compound(s) with minimum post-extraction purification steps [13]. The use of such advanced preparatory tools is likely to ensure batch-to-batch uniformity in the composition of herbal extracts, with the least variation in biological efficacy. Consequently, experimental results obtained with such extracts tend to be more reproducible and veritable.

The overall ease of dosing zebrafish (just by water dissolution or through the feed), makes it an extremely attractive pharmacological and toxicological study model system for recapitulating several human diseases and disorders, including reproductive abnormalities. Zebrafish is a very relevant in vivo model for studying human reproductive problems, due to its short reproductive cycle and a high degree of similarity with human reproductive regulatory systems, at hormonal and neuronal levels [15]. The use of N-ethyl-N-nitrosourea (ENU) by water dissolution is an accepted method for inducing fertility mutations in zebrafish at a high rate, offering a pool to screen fishes that are either totally sterile or have impaired fertility depending on the need [16]. Therefore, zebrafish was chosen as an ideal model system to investigate the effects of *Shivlingi* seed extract on fertility.

Scientific validation for the Ayurvedic practise of treating fertility problems with *Shivlingi* seeds has been provided through this study. This study can be divided into two aspects: (1) production of SLSO through SCFE and its compositional investigation through Gas Chromatography-Mass spectrometry (GC–MS/MS) and High-Performance Thin Layer Chromatography (HPTLC), and (2) its functional validation as infertility remedial using the ENU mutagenic zebrafish model of the disorder. The potential of SLSO in restoring fertility was comparable to that of commonly used infertility drugs.

## Results

### Fatty acids and phyto-sterol constituents of SLSO

**Fig. 1:**
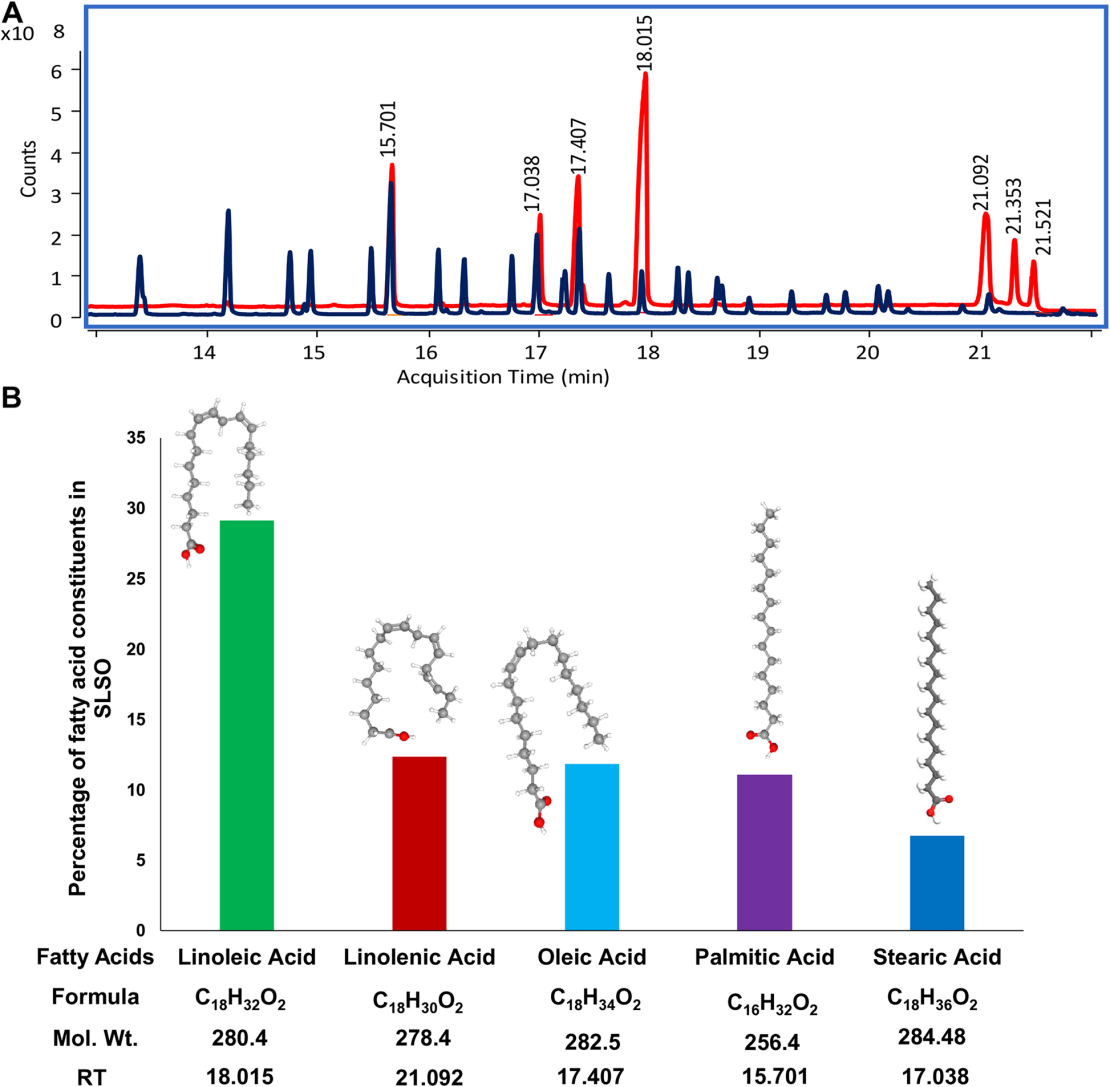
Fatty acid constituents of the SLSO and their quantity in percentage. **A** Chromatograph of standard mix FAME in blue colour and *Shivlingi* seed SCFE oil in red colour by GC–MS/MS. **B** Bar graph showing the fatty acid composition of SLSO as determined through GC–MS/MS. The chemical structures were sourced from PubChem (https://pubchem.ncbi.nlm.nih.gov/) and ChemSpider (https://www.chemspider.com/) (accessed on 1 December 2021)

Several batches of SLSO were extracted and the average yield was estimated. Oil from one such extraction was used to determine the phyto-chemical constituents and subsequent in vivo demonstration of the rescue of zebrafish with impaired fertility phenotype. The SLSO in this particular batch of extraction resulted in a yield of 65 gm oil from 1.5 kg powdered seed material (4.33% yield). This was also the yield from five different extractions. GC–MS/MS analysis of the oil using helium as a carrier led to the detection of five fatty acids; two major ones, namely, linoleic (29.13%) and linolenic (12.37%), being polyunsaturated and essential. The other fatty acids present in SLSO in lesser amounts are oleic (11.85%), palmitic (11.06%), and stearic (6.75%) acids (Fig. 1).

**Fig. 2:**
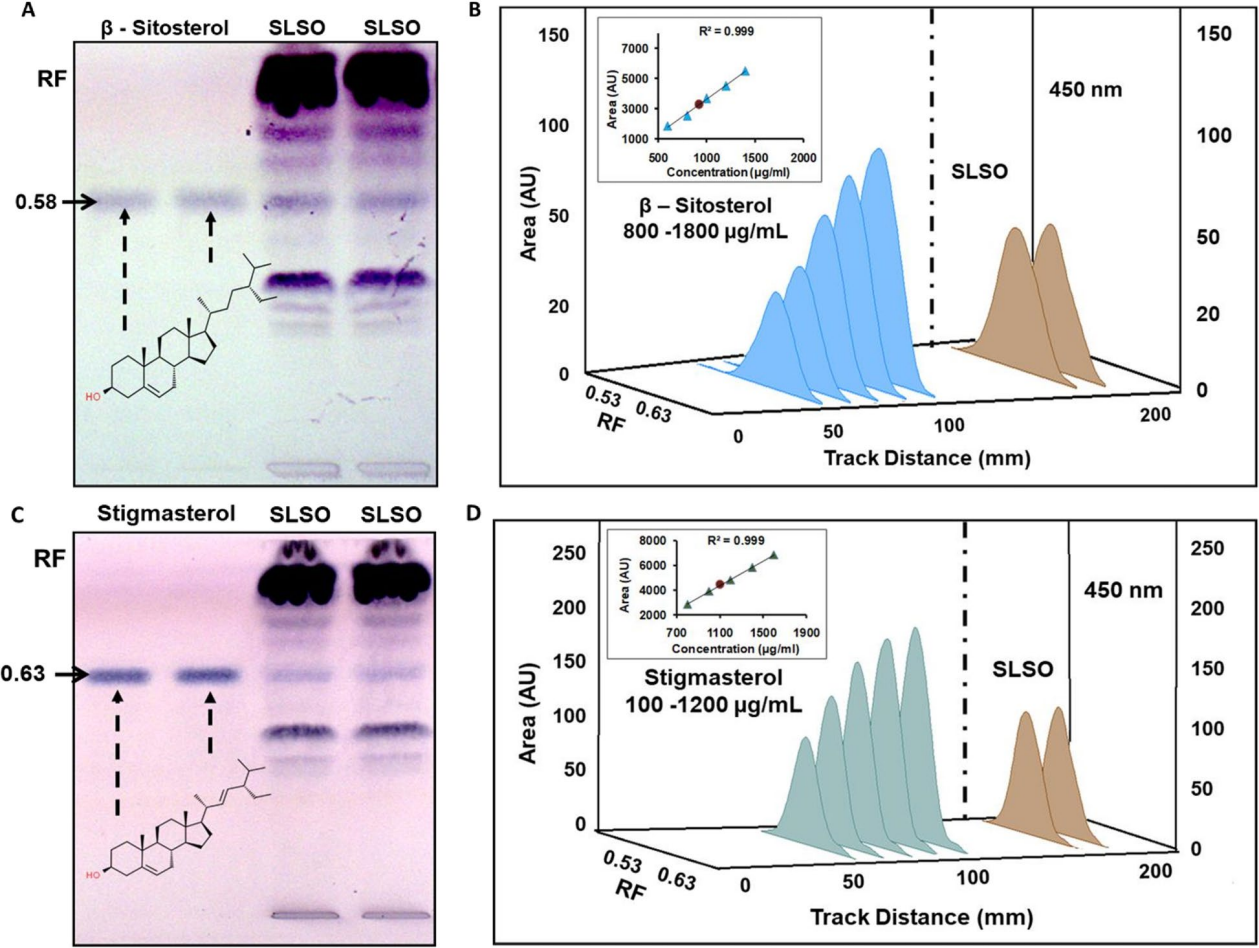
HPTLC of SLSO for identification and quantification of β-sitosterol and stigmasterol. **A**, **C** Representative images of TLC plate photographed under white light after derivatization with anisaldehyde sulphuric acid reagent. The concentrations of standard β-sitosterol in the image were 1600 and 1800 µg/mL, while for stigmasterol, they were 1000 and 1200 µg/mL. The injection volume of SLSO was 2 µL and the mobile phase used was chloroform:methanol in 9:1 ratio (v/v). **B**, **D** Known amount of oil extract of SLSO (Brown line) was compared with known concentrations of standard β-sitosterol (Sky blue lines) and stigmasterol (Dark blue lines). Duplicate loadings of SLSO were done on the same plate. Inset shows the linearity curve of the standard at various concentrations

Two phytosterols, namely, β-sitosterol (Fig. 2A) and stigmasterol (Fig. 2C), were identified in SLSO. Standards (1 µL) of β-sitosterol (90.5% purity) and 2 µL of SLSO were resolved on a silica gel TLC plate with chloroform:methanol as the mobile phase. A representative image of the TLC after derivatization with anisaldehyde sulphuric acid reagent is shown in Fig. 2A. HPTLC spectra of SLSO, scanned over 350 to 700 nm, overlapped well with that of the β-sitosterol standard, thereby, confirming the identity of the phyto-sterol (Fig. 2B). Upon estimation using a standard curve with a linearity range of 800 to 1800 µg/mL, β-sitosterol content of SLSO was found to be 1.01%. Likewise, the identity of stigmasterol in SLSO was confirmed through the HPTLC spectrum, over 350 to 700 nm, overlapping with the standard (Fig. 2D). The components of SLSO were resolved on a TLC with different concentrations of stigmasterol. The TLC plate was derivatized using anisaldehyde sulphuric acid and a densitometric scan was performed. A representative photograph of the TLC plate post-derivatization and under white light is shown in Fig. 2C. The overlay spectra of the stigmasterol band from both the standard and the SLSO sample were scanned between 350 to 700 nm. SLSO was found to contain 0.35% stigmasterol, when quantified using a standard curve in the linearity range of 100 to 1200 µg/ml.

### SLSO was found to be safe in zebrafish model of infertility

Zebrafish were treated with 5 mM ENU by water dissolution for 15 min each day for 30 consecutive days as detailed in the methods section. This protocol was modified from the existing protocol to reduce the number of deaths due to mutagenesis. A total of 550 females were exposed to the mutagen, with 542 survivors at the end of the mutagenesis period. The fish were rested for 14 days before the start of the study and were bred twice at weekly intervals during this period. Female zebrafish that produced less than 20 embryos were mated with healthy males and labelled as fertility impaired mutants (413 females).

In the case of male zebrafish, they were subjected to 100 mM metronidazole for further 20 days post-ENU treatment. Metronidazole leads to testicular cell ablation and therefore, only partial treatment was used to obtain zebrafish impaired with reversible fertility. A total of 550 fish were subjected to the mutagenesis protocol and out of the 519 survivors, 450 males were termed as fertility impaired mutants as determined by the production of less than 20 embryos upon breeding with healthy wild-type females.

**Fig. 3:**
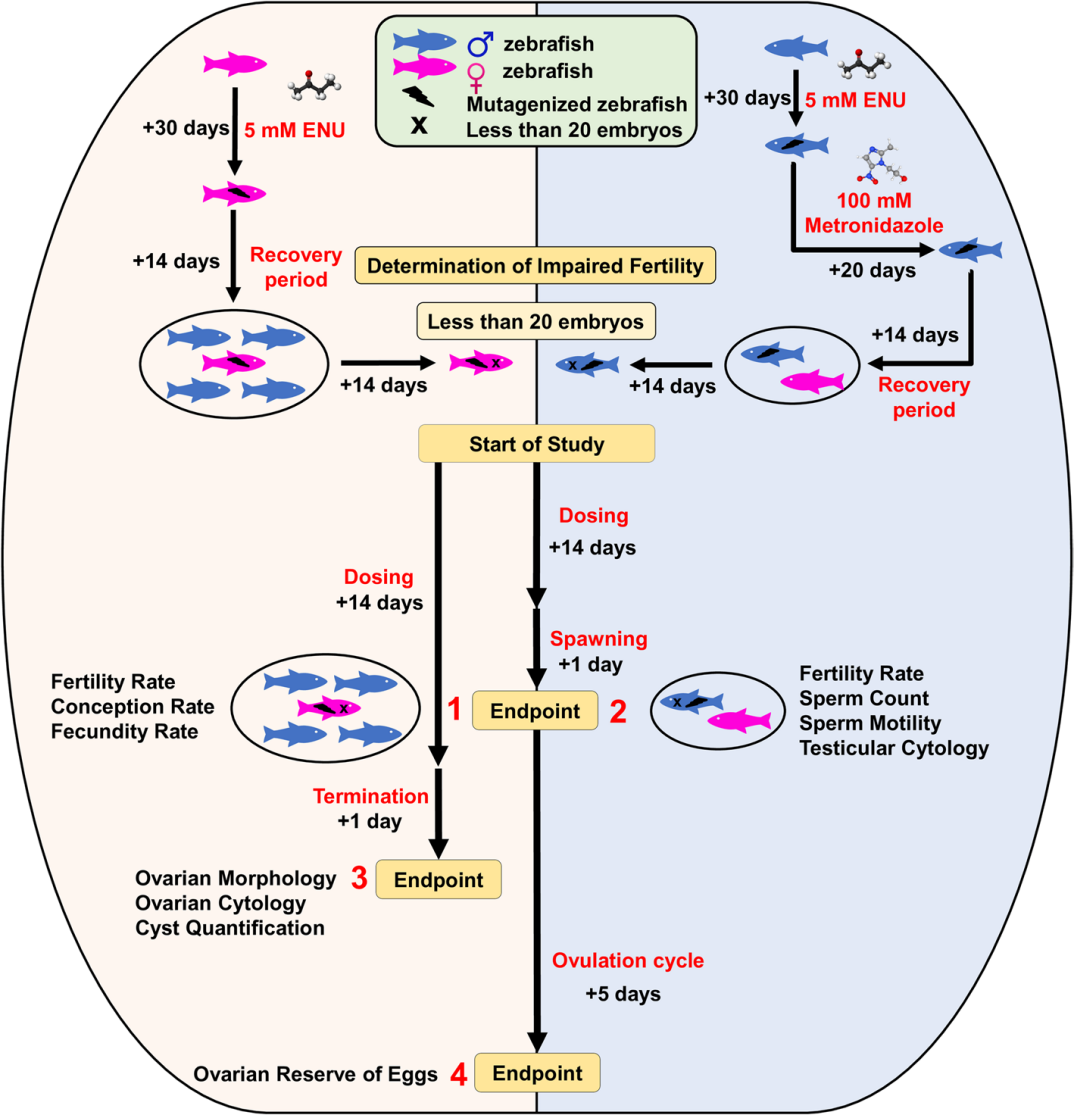
Schematic of the experimental design, with timelines and endpoints of the study. Fertility impairment was induced in the female zebrafish using only ENU mutagenesis, while in male zebrafish, ENU mutagenesis was followed by treatment with metronidazole. Mutagenized zebrafish producing less than 20 embryos were considered as fertility impaired models and used in the study. Various study groups comprising of healthy control, fertility impaired models, zebrafish treated with letrozole (female)/clomiphene (male), and zebrafish treated with six different doses of SLSO were used in the study (Fig. 4). After 14 days of dosing, the fish were allowed to spawn for 1 day, followed by data collection at endpoints 1 and 2 in females and males, respectively. In females, the ovulation cycle was allowed for the next 5 days, followed by dissection at endpoint 4 to assess the ovarian reserve of eggs. In a parallel experiment, female fish were dosed for 14 days and then dissected on the 15^th^ day to study ovarian morphology, cytology, and cyst at endpoint 3

Once the fertility impairment was induced, the fish were kept in groups of 24 individuals along with a healthy control group. The fertility impaired mutants were given a diet with either the reference drugs (letrozole for females and clomiphene for males) or with the SLSO at different dosages (Fig. 3).

Zebrafish with diets containing either the standard drugs or the SLSO did not show any mortality over the study period. The healthy control group and the fertility impaired group also did not have any mortality. This suggested that treatment with SLSO even at 100X (128 µg/kg) of the human equivalent dosage was non-toxic to the zebrafish model.

### SLSO treatment showed recovery of fertility, conception, and fecundity in the fertility impaired model

**Fig. 4:**
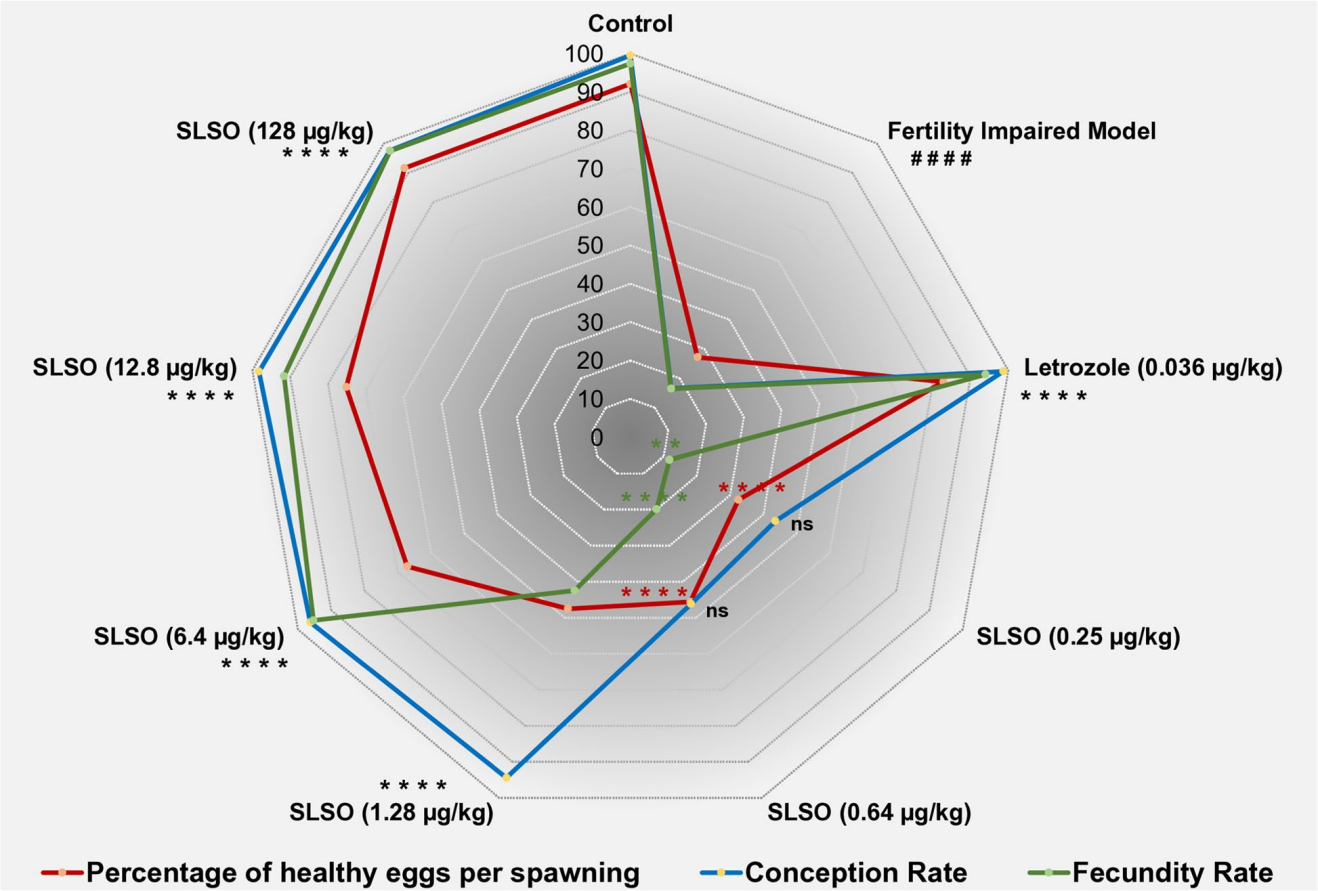
Contour plot of percentage of healthy egg per spawning, conception rate and fecundity rate in the different study groups of female zebrafish. Experiments were set with 24 breeding pairs. The percentage of healthy eggs released by the females during spawning was calculated from the total number of eggs spawned. The formation of a viable embryo represents a positive conception. The rate of conception was calculated by the number of viable embryos per positive breeding. The hatching of viable larvae from the egg was considered as a positive for the calculation of fecundity. The total number of embryos that led to viable larvae by the number of viable embryos was calculated as the rate of fecundity. Data were represented as mean ± SD (*n* = 24) and statistical significance was derived by One-way ANOVA followed by Tukey’s multiple comparison test. #### represents *p* < 0.0001 compared to the normal control group, ** represents *p* < 0.01 compared to the fertility impaired group, while **** represents *p* < 0.0001 compared to the fertility impaired group

The fertility rate in the female zebrafish study groups was calculated as the number of positive spawning events irrespective of the formation of confluent embryos to the total number of breeding pairs. The control group comprising of healthy females in a mating event with four healthy males showed a fertility rate of 100%, while the fertility impaired group had a rate of 83.3% (Fig. 4). All the treatment groups showed a fertility rate of 100%.

With a 100% fertility rate, the number of healthy eggs per spawning was 92.13% in the healthy control group. In comparison, the fertility impaired model had a significantly lower (*p* < 0.0001) percentage of healthy eggs at just 27.21% (Fig. 4). Treatment with letrozole restored the percentage of healthy eggs per spawning to 82.87% which was significantly higher (*p* < 0.0001) than that of the fertility impaired group. The SLSO at the lowest dosage (0.2X of the human equivalent dosage; 0.25 µg/kg/day) led to the production of only 32.5% healthy eggs but was still significantly higher (*p* < 0.01) when compared to the fertility impaired group. With increasing dosages of SLSO, a dose-dependent increase in the number of healthy eggs per spawning was observed as shown in Fig. 4. The human equivalent dosage (1.28 µg/kg/day) restored the egg count to 47.5% while the 100X dosage (128 µg/kg/day) restored it to near normal levels at 91.58%.

In addition to the fertility rate, the rate of conception and the fecundity rate also showed a dose-dependent increase with increasing dosages of SLSO. The conception rate was defined as the percentage of mating events that resulted in the formation of viable embryos to the total number of mating events. The healthy control group had a conception rate of 99.54% while this was significantly reduced (*p* < 0.0001) to only 16.8% in the fertility impaired group (Fig. 4). Treatment with letrozole showed a conception rate of 98.66% which was nearly equal to that of the healthy controls. Even the lowest dosage of SLSO showed a significantly higher (*p* < 0.0001) conception rate at 43.57%. The human equivalent dosage (1.28 µg/kg/day) restored the conception rate to near-normal levels (94.43%), while the other three dosages of 5X, 10X, and 100X had similarly high rates of conception.

The formation of viable larvae from embryos determines the fecundity. The healthy control group had a fecundity rate of 97.38%, compared to 16.47% in the fertility impaired group (Fig. 4). The lower dosages of SLSO, i.e., 0.2X and 0.5X, could not recover the loss of fecundity with only 11.76% and 19.87% fecundity rates. The human equivalent dosage showed a significant increase in the fecundity rate (*p* < 0.0001) but was still low at 42.47%. The other dosages improved the fecundity rate comparable to the healthy controls (Fig. 4).

### SLSO treatment enhanced ovarian reserve of eggs

**Fig. 5:**
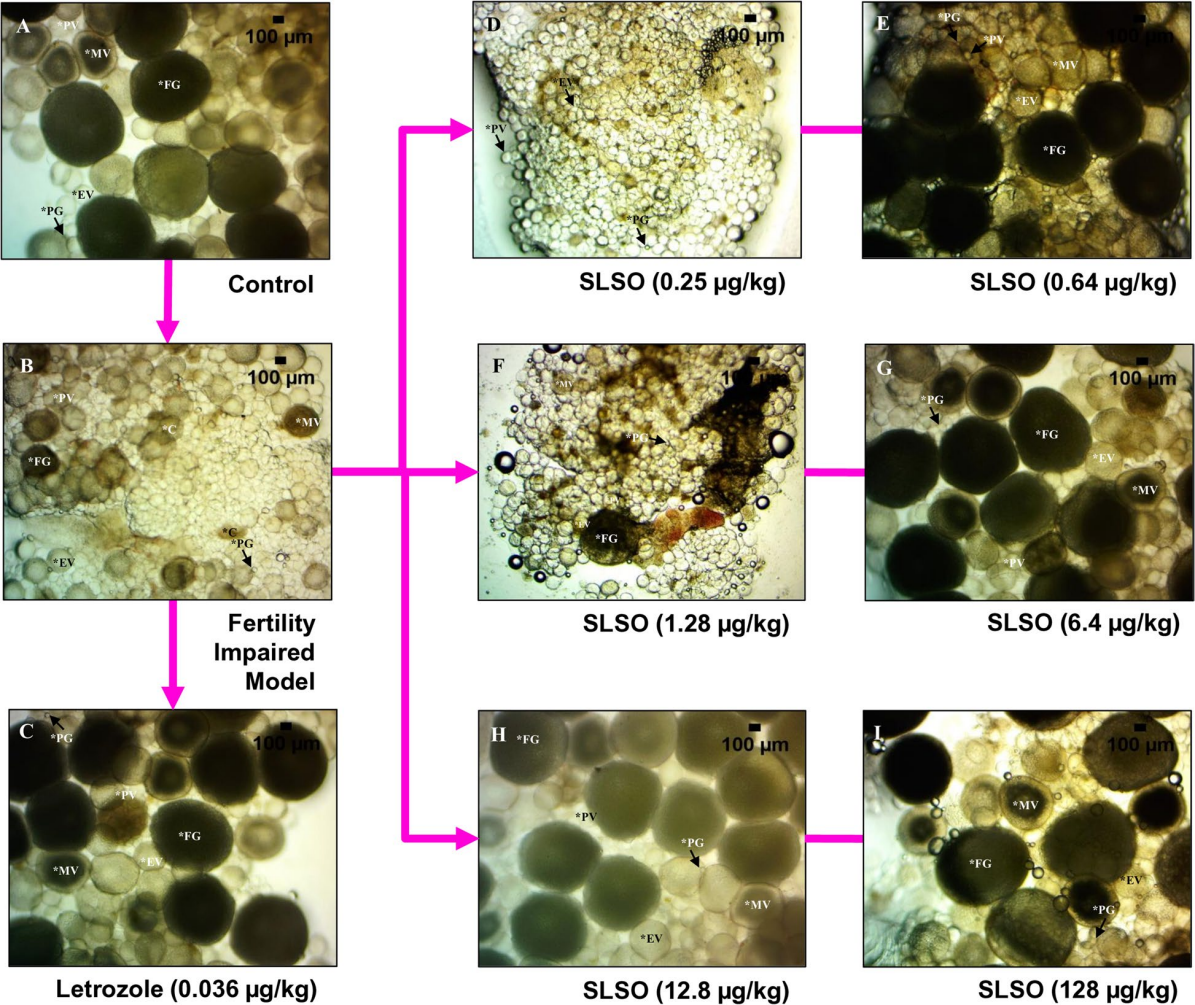
Cysts in the ovary of zebrafish from the different study groups. The cysts were identified as small dark-coloured and irregularly shaped cells that do not have a yolk. The stages of the egg development were identified and marked for reference; C- Cyst, PG- Primary Growth, PV- Pre- Vitellogenic, EV- Early Vitellogenic, MV- Mid-Vitellogenic, FG- Full Growth. The cysts were imaged under the 40X objective of a stereomicroscope

The ovarian reserve of eggs is an important factor in assessing the fertility of zebrafish. The higher the reserve of eggs, the higher is the fertility rate. In the parallel experiment, the fish were terminated on the 15^th^ day. The ovary from the fish of different groups was dissected and the follicles were gently dispersed onto a counting slide. The yolk filled eggs at different developmental stages were counted (Fig. 5). The healthy control group showed a good distribution of follicles at various stages such as Primary Growth (PG), Pre-Vitellogenic (PV), Early Vitellogenic (EV), Mid-Vitellogenic (MV), and Full Growth (FG) (Table 1). The fertility impaired group showed a significantly lower (*p* < 0.0001) number of follicles at each developmental stage as well as a lower number of total eggs in the ovarian reserve. The SLSO at the lowest dosage did not show a significant increase in the number of follicles at the PG, EV, and the MV stages but had a significantly higher (*p* < 0.01) number at the PV and FG stages. The total number of eggs in the ovarian reserve was also significantly higher (*p* < 0.0001) compared to the fertility impaired group. The human equivalent dosage had an egg reserve that was slightly higher than that in the group treated with letrozole. The follicles were predominantly in the PG and PV stages and were significantly higher (*p* < 0.0001) than that seen in the fertility impaired group. A similar distribution was also seen in the group treated with SLSO at 5X of human equivalent dose, along with an increase in the follicles at the Final Growth stage. The dosages that were 10X and 100X of the human equivalent dosage showed near-complete reversal of the fertility impairment. The total egg reserve was almost equal to that of the healthy control group and there was a good distribution of all the different developmental stages.

**Table 1:** Ovarian reserve of eggs determined by the number of ovarian follicles at different stages of development

	Study Groups	Control	Fertility impaired model	Letrozole (0.036 µg/kg)	SLSO (µg/kg)					
					**0.25**	**0.64**	**1.28**	**6.4**	**12.8**	**128**
Follicle count—Ovarian Reserve of Eggs	**Primary growth (PG)**	114 ± 8.2	97 ± 6.8^####^	137 ± 7^****^	99 ± 9.2	113 ± 8.5^****^	320 ± 6.9^****^	352 ± 5.9^****^	458 ± 7.9^****^	389 ± 9.8^****^
	**Pre-Vitellogenic (PV)**	296 ± 8.1	79 ± 8.9^####^	256 ± 7.7^****^	83 ± 8.5	94 ± 6.3^****^	490 ± 6.9^****^	291 ± 7.9^****^	125 ± 8.4^****^	131 ± 6.6^****^
	**Early Vitellogenic (EV)**	311 ± 8.3	16 ± 6.8^####^	140 ± 7.4^****^	27 ± 8.5^****^	31 ± 7.8^****^	34 ± 7.5^****^	39 ± 7.4^****^	208 ± 8.4^****^	264 ± 7.5^****^
	**Mid-Vitellogenic (MV)**	109 ± 7.8	9 ± 7.6^####^	96 ± 8.4^****^	10 ± 5.8	20 ± 6.3^****^	31 ± 8.0^****^	28 ± 7.2^****^	49 ± 7.0^****^	64 ± 9.1^****^
	**Final growth (FG)**	483 ± 7.6	12 ± 6^####^	356 ± 7.6^****^	31 ± 7.1^****^	91 ± 8.0^****^	119 ± 7.7^****^	196 ± 8.4^****^	321 ± 8.6^****^	401 ± 7.8^****^
	**Total**	1313 ± 16.3	213 ± 15.3^####^	985 ± 18.0^****^	250 ± 17.0^****^	348 ± 18.4^****^	993 ± 15.6^****^	906 ± 18.4^****^	1161 ± 17.6^****^	1249 ± 19.0^****^

Data represented as mean ± SD (*n* = 24). Statistical significance was arrived at using ANOVA followed by Tukey’s multiple comparison test

^####^ represents *p* < 0.0001 compared to normal control, **** represents *p* < 0.0001 compared to the fertility impaired group

### SLSO treatment reduced ovarian cysts

**Fig. 6:**
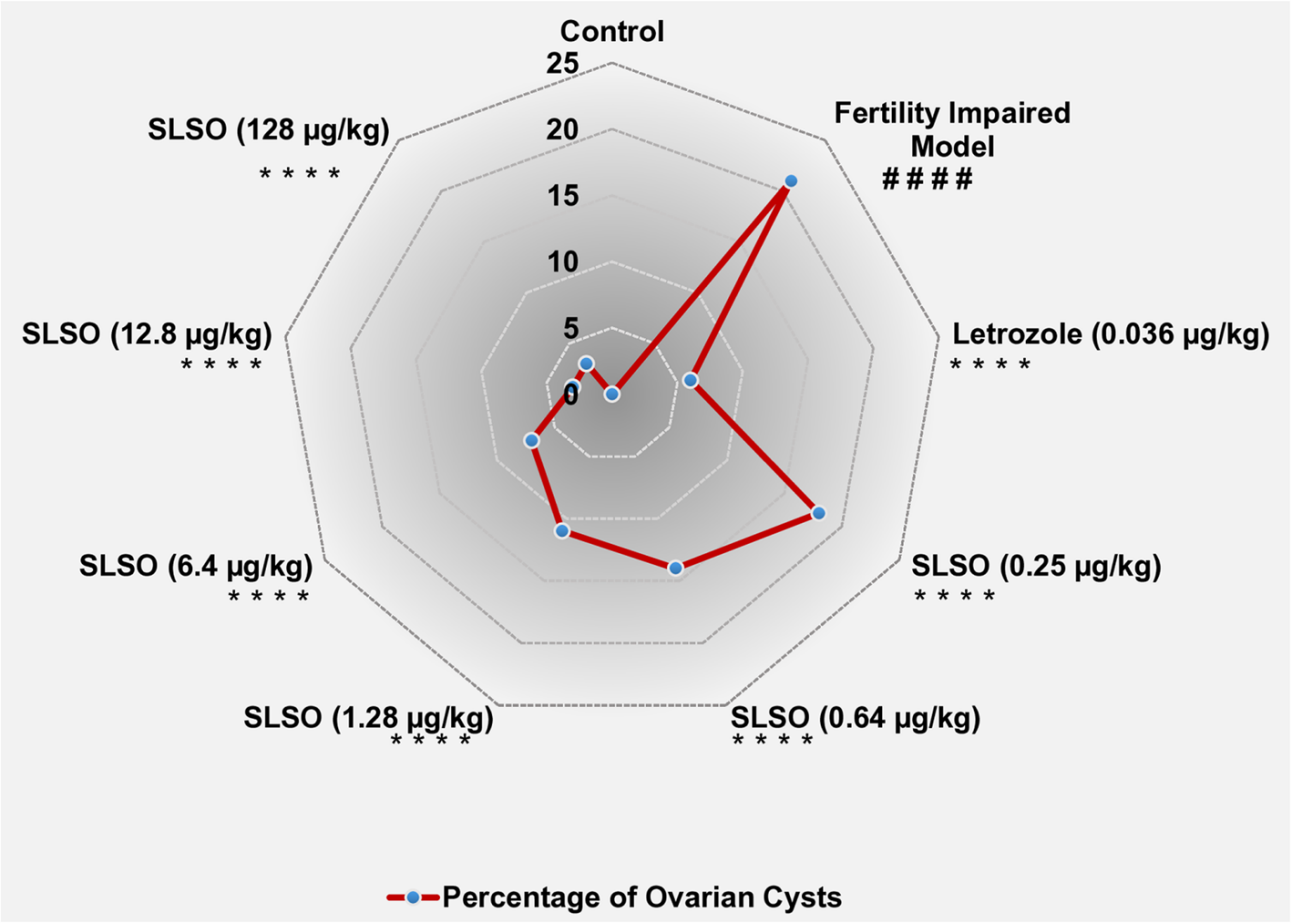
Contour graph of the number of cysts from each study group. Data are represented as mean ± SD and One-way ANOVA followed by Tukey’s multiple comparisons test to determine statistical significance. ## represents *p* < 0.01 compared to the healthy control group, ####represents *p* < 0.0001 compared to the healthy control group, **** represents *p* < 0.0001 compared to the fertility impaired group, *n* = 24

Cysts in the ovary are identified as irregularly shaped cells, dark in colour and filled with fluid rather than the yellowish yolk. The covering of the cyst is also very thin, which ruptures easily during handling. The ovary was carefully dissected and observed under the 40X objective of a stereomicroscope to quantify the number of cysts present in the different study groups. No cysts were identified in the healthy control group, while the fertility impaired model showed a significantly higher (*p* < 0.0001) number of cysts (Fig. 6). When quantified, 21% of the follicles observed from the ovary were cysts. Treatment with letrozole significantly reduced the number of cysts in the ovary but was unable to totally rescue the phenotype, as about 6% were still in the form of cysts. The lowest dosage of SLSO resulted in a significant reduction (*p* < 0.0001) in the number of cysts compared to the fertility impaired group, but the mean percentage of cysts in this group was still about 18%. Although treatment with higher dosages showed a dose-dependent reduction in the percentage of cysts, treatment with the human equivalent dosage (1.28 µg/kg/day) still had about 11% cysts. Dosages at 10X and 100X showed maximum reversal of the phenotype with only 3% of the total ovarian follicles determined to be cysts.

### SLSO caused recovery in anatomical features of ovary in fertility impaired model

**Fig. 7:**
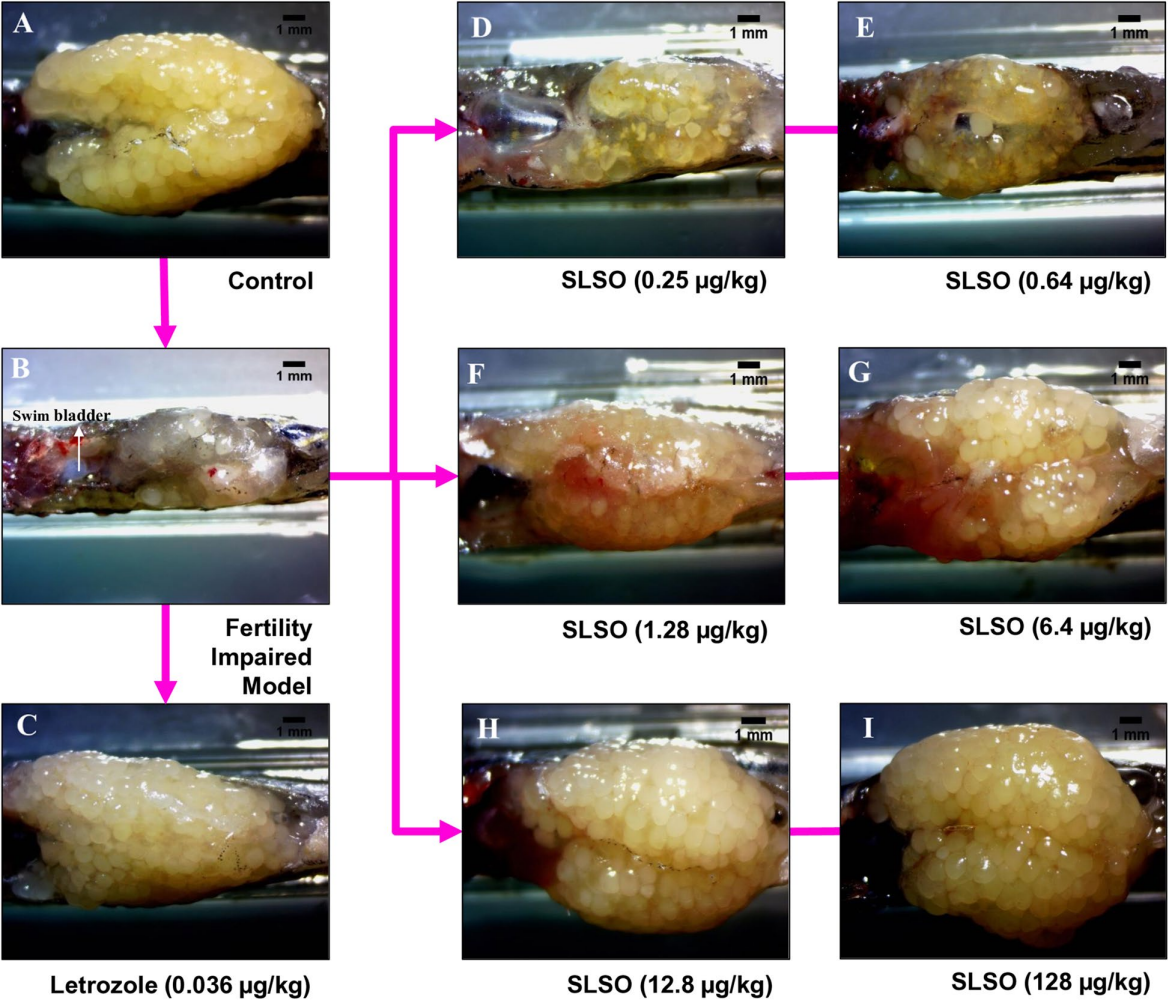
SLSO-induced recovery in the gross morphological features of the ovary in the various study groups. Representative images were captured with a stereo-microscope using a 10X bright field objective. Letrozole was used at 0.036 µg/kg/day. The different dosages of SLSO were represented with reference to the human equivalent dosage; 0.2X (0.25 µg/kg/day), 0.5X (6.4 µg/kg/day), 1X (1.28 µg/kg/day), 5X (6.4 µg/kg/day), 10 X (12.8 µg/kg/day), and 100X (128 µg/kg/day)

In a parallel experiment, fertility impaired female fish were dosed for 14 days and dissected on the 15^th^ day for analysis of the gross anatomy to determine the overall volume and health of the ovary. Ovary from the control group showed a voluminous gonad bristling with eggs at various developmental stages and no visible signs of haemorrhage (Fig. 7). The ovary isolated from the fertility impaired model was less voluminous compared to the control group and had a lesser number of follicles that appeared to be immature. A few follicular cysts were also observed that were filled with pale-coloured fluid and ruptured easily upon handling. Ovaries were dissected from all the zebrafish in the study groups and there were negligible differences in the overall appearance and volume. The group treated with letrozole had well-defined ovaries with a significantly higher number of follicles compared to the fertility impaired group. The groups treated with SLSO showed a dose-dependent increase in the volume of the ovary. Though the ovary was comparatively less voluminous in the groups treated with 0.25, 0.64, and 1.28 µg/kg/day SLSO compared to the healthy control group, it was comparatively better than that seen in the fertility impaired group. Moreover, the ovaries had loosely packed follicles within the ovary sac suggesting that the dosage was not enough to totally rescue the impaired fertility. Treatment with higher dosages (6.4, 12.8, and 128 µg/kg/day) showed an ovarian morphology that was comparable to that of the healthy control group. The follicles were identified to be from different developmental stages and showed a good numerical distribution.

### SLSO treatment restored male fertility rate and sperm count to near normal levels

**Fig. 8:**
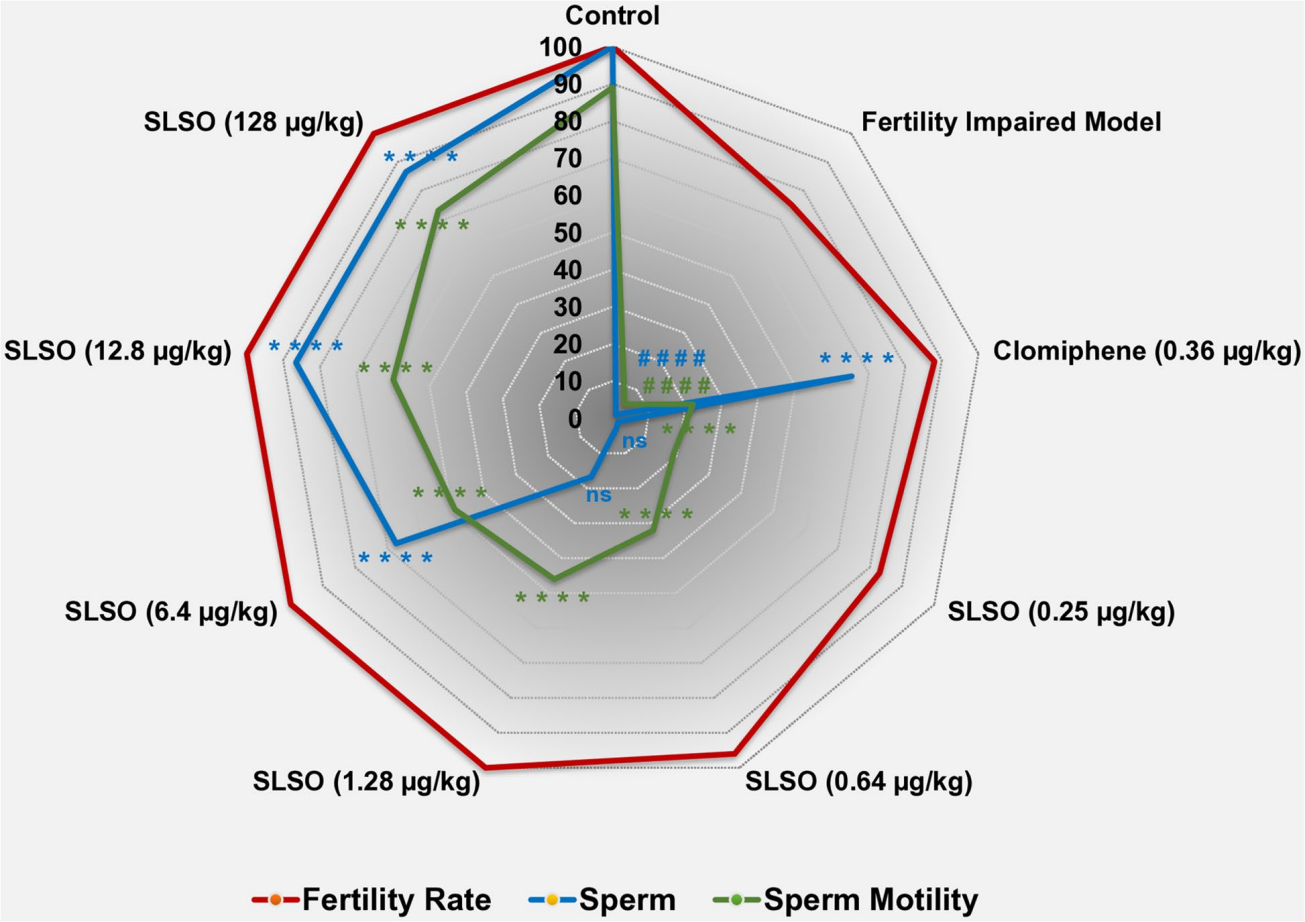
Contour graph of restoration of the fertility rate, sperm count, and sperm motility upon treatment with SLSO. The fertility rate was determined as the percentage of positive spawning events compared to the total number of breeding pairs (*n* = 24). The number of sperm per mL of ejaculate was determined and represented as percentage with respect to the control. The percentage of motility of sperm from the various study groups was determined from 1 X 10^8^ sperm using the wrMTrck plugin of ImageJ software. Data are represented as mean ± SD from 24 individuals and One-way ANOVA was used to determine statistical significance. #### represents *p* < 0.0001 compared to the healthy control group, and **** represents *p* < 0.0001 compared to the fertility impaired group

Each mutant male zebrafish was set up for spawning with a single female to determine the fertility rate based on a positive spawning and formation of an embryo. Compared to the healthy control group, the fertility impaired model showed only 75% positive breeding out of the 24 breeding pairs (Fig. 8). Treatment with clomiphene was able to restore the fertility rate to only 88%. In contrast, the SLSO at human equivalent dosage was able to completely restore the fertility rate to 100% similar to that of the healthy control group.

The lower rate of fertility in the fertility impaired group could be explained by the significantly lower (*p* < 0.0001) sperm count compared to the healthy control (Fig. 8). Moreover, the sperm count showed a great degree of variation between individual zebrafish in the group (Table 2). The group treated with a human equivalent dosage of clomiphene showed a statistically significant increase (*p* < 0.0001) in the number of sperm compared to the fertility impaired group. However, individuals of the group showed a great degree of variability in the sperm count, similar to that seen in the fertility impaired group. Treatment with SLSO at dosages less than the human equivalent dosage showed a modest but non-significant increase in the sperm count compared to the fertility impaired group (Table 2). Treatment with higher dosages showed a dose-dependent recovery in the sperm count, with the human equivalent dosage showing a highly significant increase (*p* < 0.0001) in the sperm count compared to the fertility impaired group, but this number was only 16.5% of the sperm count in the healthy control group. Importantly, there was very little variation in the sperm count among the individual members of the group suggesting the robustness of the treatment response. Treatment with 10X and 100X human equivalent dosage restored the sperm count to levels that were almost equal to that of the healthy control group.

**Table 2:** Sperm count and motility in various study groups

Study groups	Sperm count	Sperm motility (%)
Control	526.9 × 10^5^ ± 9.8	89 ± 2.3
Fertility impaired group	6.7 × 10^5^ ± 3.8 × 10^5^	5 ± 3.3
Letrozole (0.036 µg/kg)	341 × 10^5^ ± 128.8 × 10^5^	21 ± 9.5
SLSO (0.25 µg/kg)	9.1 × 10^5^ ± 4.0 × 10^5^	19 ± 10.2
SLSO (0.64 µg/kg)	15.6 × 10^5^ ± 3.2 × 10^5^	32 ± 7.5
SLSO (1.28 µg/kg)	87.2 × 10^5^ ± 9.5	46 ± 5.0
SLSO (6.4 µg/kg)	358.5 × 10^5^ ± 8.9	49 ± 7.5
SLSO (12.8 µg/kg)	454.5 × 10^5^ ± 8.6	60 ± 7.8
SLSO (128 µg/kg)	459.4 × 10^5^ ± 10.2	73 ± 7.0

Data represented as mean ± SD (*n* = 24)

A drastic reduction in sperm motility was observed in the fertility impaired model compared to the healthy controls (Fig. 7) and this reduction was statistically significant (*p* < 0.0001). Treatment with clomiphene increased sperm motility significantly (*p* < 0.0001) compared to the fertility impaired model; however, this increase was only 21%. The lowest dosage at 0.2X (0.25 µg/kg/day) significantly increased the motility and was comparable to that of the healthy reference group. A dose-dependent increase in sperm motility was observed with an increase in the dosage of SLSO. The human equivalent dosage showed 46% motility which was a much better response compared to clomiphene and this increase was statistically significant when (*p* < 0.0001) compared to both the fertility impaired group as well as the clomiphene treated group. The highest dosage of SLSO (128 µg/kg/day) at 100X of the human equivalent dosage reversed the sperm motility to about 73%, but still lower than the healthy control group. However, this reversal was highly significant when compared to the fertility impaired group.

### SLSO treatment restorated impaired spermatogenesis

**Fig. 9:**
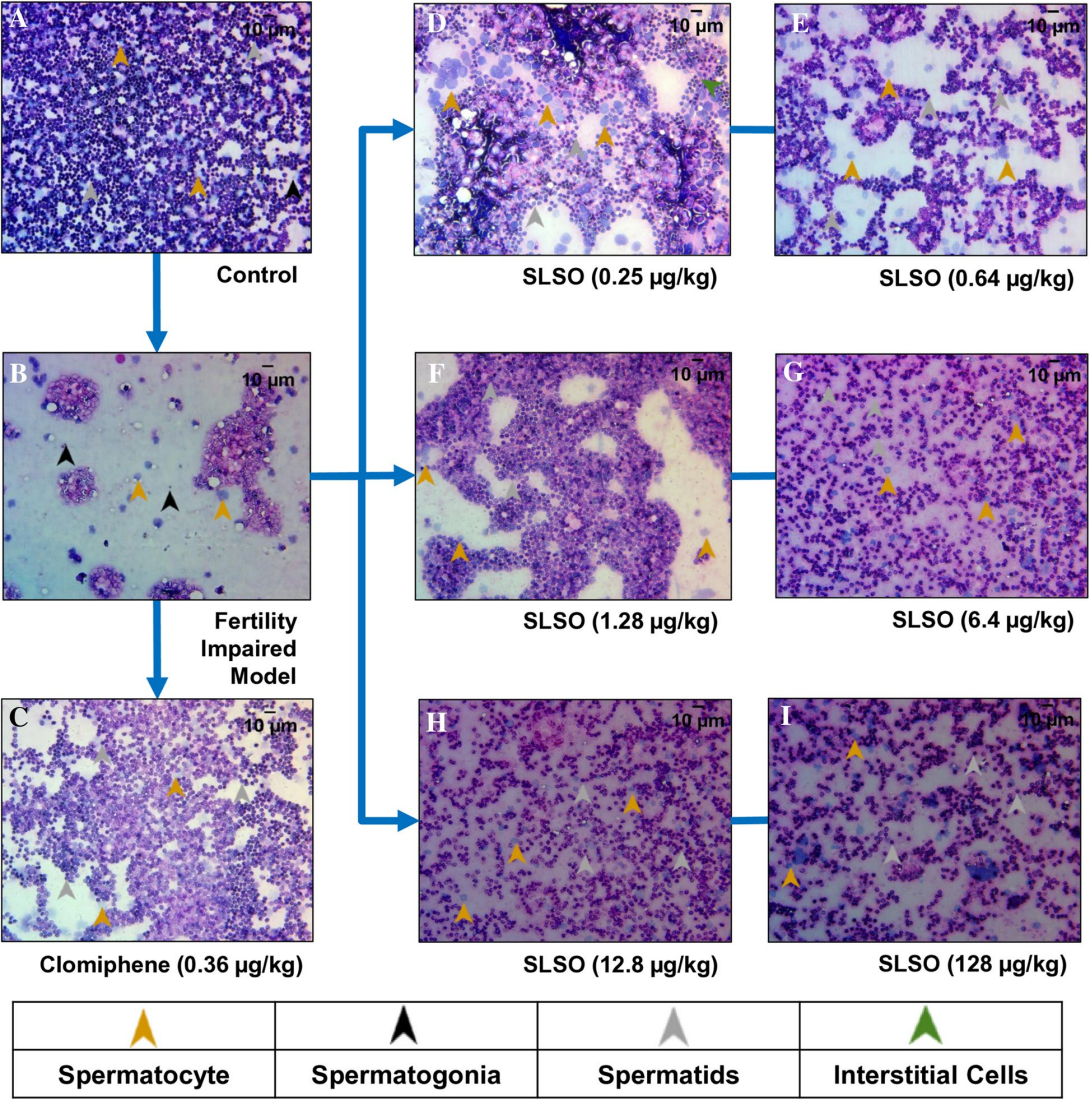
Cytology smears of testis isolated from the various study groups. Hematoxylin–eosin staining was used to assess anatomical recovery upon treatment with SLSO. Representative images captured using a 40X objective lens are shown. The images were used to identify the different developmental stages of the spermatozoa

The testis was dissected from the male zebrafish from all the different groups in the study and a cytological smear was made. The tissue was stained with hematoxylin–eosin and used to study the tissue architecture. The testis from the healthy control group was observed to have a good distribution of sperm covering all the different stages of maturation such as spermatogonia, primary spermatocytes, secondary spermatocytes, spermatids, and spermatozoa (Fig. 9). The nuclear staining showed a well-defined nucleus and eosinophilic cytoplasm, characteristics of a fully functional and mature organ. Cytology from the fertility impaired model showed a remarkably lower number of spermatozoa indicating a poor maturation cycle. In addition, a higher proportion of spermatogonia and a lower number of spermatocytes were observed. The treatment group with clomiphene showed a reversal of the impaired fertility phenotype as determined by the presence of spermatogonia at various stages of maturity. Treatment with the lowest dosage of SLSO showed an equal distribution of enlarged spermatogonia and spermatids which indicated a mildly compromised but functional gonad. Disorganized interstitial cells were also observed further suggesting that the lowest dosage was not enough to restore the gonadal function. Treatment with the human equivalent dosage showed good recovery of testicular architecture compared to the fertility impaired group. A minuscule number of spermatogonia cells were noted and an elevated number of spermatids were observed. Treatment with higher than the human equivalent dosages showed a higher proportion of spermatids in the cytological smears suggesting complete recovery of the testes comparable to the healthy control group.

## Discussion

The seeds of *Shivlingi* are used in the Ayurvedic system of medicine as a general virility booster and are often used to treat sexual dysfunction and impaired fertility. The current work was undertaken to evaluate the scientific basis for such claims by chemically analyzing the supercritical fluid extracted SLSO and evaluating its reproductive fecundity in a zebrafish model of N-ethyl-N-nitrosourea-induced infertility.

High quantities of linoleic (47.8%) and linolenic acid (28.9%) were found in the SLSO. Fertility impaired female and male zebrafish showed improvement in fertility rates when fed with a diet rich in SLSO. In female zebrafish models, a dose-dependent increase in the follicular reserve of eggs was observed, along with a significant decrease in the number of cysts in the ovary. Similarly, in male zebrafish models, a significant increase was observed not only in the sperm count but also in the sperm motility. Moreover, gross improvement in the reproductive organs was observed, both in terms of morphological as well as cytological features. Microscopic studies revealed improvement in ovarian morphology with increased dosage of SLSO. Accordingly, the number of cysts and inflammatory cells also reduced in fertility impaired zebrafish as is evident from cytological studies of the ovaries. Similarly, in zebrafish male models, the cytological studies of the testis confirmed improvement in testicular health with healthy spermatogonia at various stages of maturity. These results establish the role of SLSO-rich diet in reversing the phenotype associated with impaired fertility in both males and females.

Polyunsaturated fatty acids such as linoleic and linolenic acids are known to enhance follicle growth in cattle [17]. They also modulate cholesterol biosynthesis, cellular metabolism, and steroidogenesis among other essential functions in the human body. In addition, linoleic acid is the most abundant fatty acid in the follicular fluid that surrounds the developing egg and has been shown to help in maintaining the integrity of the germinal vesicles during oogenesis [18]. Studies in male white leghorn chicken showed significantly reduced average fertility when fed a diet deficient in linoleic acid [19]. In a randomized clinical trial, a group of men given Omega-3 supplements had increased sperm count as well as sperm density. One of the reasons attributed to this was the reduction in the reactive oxidative species (ROS) levels in the sperm. In humans, a strong positive correlation has been observed between sperm motility and sperm membrane docosahexaenoic acid (DHA) levels, which in turn is synthesized from α-linolenic acid [20]. Low quantities of oleic acid enhanced the shelf-life of rooster semen during cold storage [21]. Studies suggest that oleic acid can contribute to female reproduction through the normal development of oocytes and preimplantation embryos [21]. These studies highlight the role of linoleic, linolenic and oleic acids in enhancing fertility in both males and females, which correlates very well with the observations from the current study. However, palmitic and stearic acids are saturated fatty acids and contribute to lipotoxicity [22].

The SLSO was also found to contain β-sitosterol and stigmasterol. Since stigmasterol is a precursor in the synthesis of β-sitosterol and shares structural similarity with cholesterol, it is quite possible that it may undergo oxidation by free radical mediated pathways such as those documented for cholesterol [23]. However, the existence of this oxidation pathway in plants is not yet known. A high concentration of β-sitosterol was not found to have any adverse effects on the reproductive rate of Fathead Minnow (*Pimephales promelas*) [24]. In fact, β-sitosterol has been shown to improve urinary flow in patients with Benign Prostate Hyperplasia (BPH). Although the reasons for this beneficial effect of β-sitosterol in BPH are not yet known, it has been speculated that it may work through cholesterol metabolism or by reducing inflammation [25]. Studies on stigmasterol by the European Food Safety Authority did not find any adverse effect on fertility and reproductive functions, such as sexual maturity, oestrous cycle, and histopathology of reproductive tissue in healthy rats [26]. Moreover, stigmasterol has been identified to have a role in cholesterol metabolism and may play a role in the inhibition of several pro-inflammatory cytokines and matrix-degrading proteases in osteoarthritis [27]. In addition, it has been found to have a pro-oxidative effect in mice in a thyroid dysfunction model [28].

## Conclusion

The fatty acid constituents of SLSO, linoleic and linolenic acids, along with the phyto-sterols, β-sitosterol and stigmasterol, have known effects on cholesterol metabolism, oxidative stress, and inflammation. In this study, the rescue of fertility impairment in ENU-mutagenized zebrafish models was observed. There was a significant increase in the fertility rate and fecundity of female zebrafish treated with the SLSO. A dramatic increase in the number of follicles at different developmental stages was also observed. These results are a reflection of the beneficial effects of SLSO on ovarian morphology and cytology. Similarly, fertility impaired males treated with SLSO showed a rescue of testicular anatomy evident through a significant increase in sperm count as well as sperm motility. Overall, this study demonstrated for the first time the fertility restoring effects of Super Critical Fluid extracted *Shivlingi* seed oil in fertility impaired zebrafish models. Apart from providing scientific validation to Ayurvedic treatment, a solid groundwork is established for further research in this area. Further studies to decipher the possible pathway(s) by which the phyto-constituents of *Shivlingi* seeds rescue impaired fertility are needed to develop this classical Ayurvedic treatment into a therapeutic for impaired fertility in humans.

## Materials and methods

### Chemicals and reagents

All the chemicals used in the study were of analytical grade (AR) and were procured from Sigma-Aldrich (St. Louis, MO, USA) unless specified otherwise. Commonly used reagents such as PBS pH 7.4 and embryo buffer were made in house according to accepted methods.

### Super critical fluid extraction of oil from *Shivlingi* seeds

Fully matured seeds of *Bryonopsis laciniosa* (L.) Naud. (*Shivlingi*) were procured from Divya Pharmacy, Haridwar, India. These seeds were further evaluated by our in-house botanists Dr. Rajesh Mishra and Acharya Balkrishna and authenticated for purity. The seeds from five different batches were used for extraction and oil from one such batch (Batch number D4/CHM/SCFE15510919) was used for the chemical analyses as well as in vivo experiments in this study.

The extraction process was accomplished using Super Critical Carbon dioxide (SC-CO_2_). Briefly, the seeds of *Shivlingi* were pulverized into a fine powder and 1.5 kg of this powder was used to extract the oil in a supercritical extractor SFE 5000 Bio-Botanical Extraction System (Waters Corporation, Milford, MA, USA). The extractor was equipped with a CO_2_ recycler and the backflow pressure was maintained at 450/80 and 55/4 Bar/°C and a CO_2_ flow rate of 70 g/min over a period of 480 min. The initial extraction was in the static phase followed by a dynamic phase extraction to get a yield of 65 gm oil. This puts the yield of oil from this batch at 4.33% and was also the average yield from several other batches of extraction.

### Identification and quantification of fatty acid constituents in super critical fluid extracted oil from *Shivlingi* (SLSO)

The fatty acid content in SLSO was determined according to the accepted method (AOCS Official Method Ce 2–66) of the American Chemist’s Society [29]. Briefly, the SLSO was mixed with 0.5 N methanolic NaOH and heated for 10 min to create fat globules. After this, BF_3_-methanol was added to the boiling mixture and was further heated for 2 min. To this, n-heptane was added and boiled for a minute. The mixture was removed from heat and saturated NaOH solution was added, followed by vigorous mixing. After allowing the mixture to cool for 10 min at room temperature, the top layer of n-heptane containing fatty acid mixture called the Fatty Acid Methyl Ester (FAME) was collected.

GC–MS/MS (7000D GC/MS triple quad with 7890B GC system, Agilent) was used to analyse the fatty acid mixture in the FAME using helium as a carrier. A standard mix for C4-C24 FAME containing a mixture of 37 FAMEs (Sigma-Aldrich, St. Louis, MO, USA) was used to ascertain the identities of the fatty acids present in the SLSO.

### HPTLC for identification and quantification of β-sitosterol in SLSO

Briefly, 500 mg sample was dissolved in 5 mL of chloroform. It was sonicated in a Fisherbrand™ Model 505 sonicator (Fisher Scientific, Hampton, NH, USA) for 20 min on ice to obtain a clear solution. The sample was centrifuged at 8000 rpm for 5 min to remove the insoluble material. The supernatant was transferred to a sterile volumetric flask and the volume made up to 10 mL with chloroform. A standard stock solution was prepared by dissolving 10 mg of β-sitosterol (90.5% purity, Natural Remedies, Bangalore, Karnataka, India) in 1 mL methanol. Working solutions were prepared by further dilutions as required.

Standard β-sitosterol (1 µL) at different concentrations (800 to 1800 µg/mL) and SLSO sample (2 µL) were spotted on an aluminium backed TLC plate with silica gel 60 F_254_ (Merck Millipore KGaA, Darmstadt, Germany). The different phyto-constituents were resolved on the plate using a mobile phase consisting of chloroform and methanol in a ratio of 9:1, which was finalized after many trials during the protocol development stage. After resolution, the bands were developed upon derivatization using anisaldehyde sulphuric acid reagent and photographed under white light. Densitometric scanning was performed using the derivatized TLC plate at 450 nm. The concentration of β-sitosterol in SLSO was estimated using a standard curve of known concentrations of the standard and compared with duplicate loadings of the SLSO sample.

### Identification and quantification of stigmasterol in SLSO by HPTLC

HPTLC analysis was done for the identification and quantification of stigmasterol in SLSO. The SLSO sample solution for HPTLC was prepared as described above by dissolving 1 g of SLSO in a total of 10 mL chloroform. A standard stock solution of stigmasterol (95% purity, Sisco Research Laboratories Pvt. Ltd., Mumbai, Maharashtra, India) was prepared at a concentration of 10 mg/mL in methanol. It was further diluted as appropriate to get standard working solutions of 100 to 1200 µg/mL. A TLC plate with aluminium backing coated with Silica gel 60 F254 was used to resolve 1 µL of standard stigmasterol (100 to 1200 µg/mL concentrations) and 2 µL of SLSO sample. The bands were resolved and derivatized as described above.

### In vivo study to determine the effect of SLSO in reversing the fertility impairment in an ENU mutagenesis zebrafish model

#### Animal ethics

The Committee for the Purpose of Control and Supervision of Experiments on Animals (CPCSEA), Government of India, guidelines were followed throughout the study period. All animal handling was done ethically to minimize stress to the zebrafish and all the protocols were duly submitted and approved by the Institutional Animal Ethics committee of Patanjali Research Institute, Haridwar (vide protocol number: 216/Go092019/IAEC).

#### Rearing of zebrafish and husbandry

Wild-type zebrafish (*Danio rerio*) of AB strain, from a single breeding pair, were used in the study to generate fertility impaired mutants. The fish were reared in groups of 24 with 2 L of water per tank in sturdy polypropylene tanks that meet the CPCSEA guidelines. The water temperature was maintained at 27 °C ± 1 °C using automated temperature control and water circulation systems. The tanks were checked daily for left-over food particles that were not cleared by the filtration system to prevent unwanted growth in the tanks. Aged water (for de-chlorination) was used and pH was routinely checked and adjusted if needed. The water was replenished as needed and full water exchange was done every week.

#### Induction of fertility impairment in female zebrafish

Fertility impairment in the female zebrafish was induced with N-ethyl-N-nitrosourea (ENU). The accepted mode of induction can cause a higher rate of mortality in the fish and therefore, the protocol was modified to suit the needs of the study [16, 30, 31]. Adult fish were exposed to 5 mM ENU dissolved in water for 15 min and then transferred back to normal water. This exposure routine was followed for 30 days. To determine the effectiveness of mutagenesis, six fish were randomly selected and were dissected to isolate the ovary. Successful mutagenesis was identified by morphological observation, where the ovary was less voluminous and by cytology, where a significantly lesser number of yolk-filled follicles were observed.

After the mutagenesis, the fish were allowed to recover and during this time, they were used in mating experiments with wild-type males (1:4 ratio of female to male) twice, under standard conditions. In our experience, this ratio provided the best rates of positive breeding and therefore, was used throughout the study. The number of embryos, irrespective of their viability, was determined for each breeding pair and only females that produced less than 20 embryos were termed as ‘fertility impaired females’ and were included in the study. Fish were randomly assigned into groups of 24 individuals each, while un-mutagenized fish from the same breeding pair was used as a healthy or normal control group.

#### Induction of fertility impairment in male zebrafish

In addition to ENU mutagenesis, the male zebrafish were further treated with 100 mM metronidazole in the tank water for further 20 days. Metronidazole is known to cause testicular cell ablation and therefore, a complete treatment protocol, which generally results in sterile individuals, was not used [32, 33]. Six mutagenized zebrafish were randomly selected and the testis dissected out. The morphology and cytology were determined to establish successful mutagenesis. The mutagenized male fish were used in spawning with wild-type females in a 1:1 ratio and the male fish that produced less than 20 embryos were selected as ‘fertility Impaired males’ and used in the study.

### Study design

Once the study population was selected, groups with 24 individuals each were moved to tanks designated to hold them during the study period and were acclimatized for 7 days. Fish were fed with normal Tetrabit flakes (a complete pet food for tropical fish from Tetra GmbH, Herrenteich Germany), using a 24 h feeding cycle until the start of the experimental period.

### Preparation of feed for the treatment groups and dosing

The Ayurvedic texts recommend the consumption of 2 gm of *Shivlingi* seeds per day. In our studies, the average yield of oil from the seeds is 4.33%. The equivalent dosage of oil extract for humans was calculated based on the amount of seeds to be consumed and the yield of oil and was determined to be 90 mg/day.

The translational dosage for the zebrafish was calculated according to the accepted guidelines of USFDA [34, 35]. In the study, letrozole (2.5 mg/day) was used as a reference drug for female infertility, while clomiphene (25 mg/day) was used for the male infertility model. SLSO was used at different dosages calculated as 0.2X, 0.5X, 1X, 5X, 10X, and 100X of the human equivalent dosage. The translational dosage used for the study is shown in Table 3.

**Table 3:** Translational dose of standard drug and SLSO derived from human equivalent dose per day

Study groups	Standard drug			SLSO						
	**Drug**	**Human (mg/day)**	**Translational Dose (µg/kg)**	**Human (mg/day)**	**Translational Dose/day (µg/kg)**					
					**0.2X**	**0.5X**	**1X**	**5X**	**10X**	**100X**
**Male**	Clomiphene	25	0.36	90	0.25	0.64	1.28	6.4	12.8	128
**Female**	Letrozole	2.5	0.036	90	0.25	0.64	1.28	6.4	12.8	128

The test and reference compounds were mixed with the normal feed at different dosages based on the known amount of feed. The feed was finely ground using a mortar and pestle and mixed with the compounds before being extruded out as pellets of 4 mg each. A fixed number of pellets with the target dose was given to the fish per day to achieve the required dosing. The study groups were dosed with either the reference drug or the SLSO in case of the treatment groups, and with normal feed in case of control and the fertility impaired groups.

### Study end-points for female zebrafish

The dosing was for 14 days and on the 15^th^ day, the different study end-points were analysed. Female fish in the different study groups were allowed to spawn with wild-type males (1:4 ratio between females and males) and the fertility rate, conception rate, and fecundity rate were assessed. The study groups were returned to their respective tanks and after 5 days (next ovulation cycle), the ovary was dissected and the follicle count for the ovarian reserve of eggs was estimated. In a parallel experiment, the same dosing protocol was followed and on the 15^th^ day, the ovary was dissected to determine the general morphology and cytology of the ovary as well as for the determination of the number of cysts.

### Fertility rate

A positive spawning that resulted in the formation of embryos, irrespective of their viability was used to calculate the fertility rate. The fertility rate was calculated from the number of positive mating to the total number of breeding pairs (24 breeding pairs).

Fertility Rate = (Number of Positive breeding/ Breeding pair setup) × 100.

### Conception rate

Conception affirms that fertilization has taken place and highly confluent viable embryos are present. The percentage of fertilized eggs per mating was calculated based on the number of highly confluent viable embryos to the total number of breeding pairs.

Conception Rate = (Total number of fertile embryos/ Total number of positive breeding) × 100.

### Fecundity rate

Embryos collected from the breeding tank were transferred to a 30 mL embryo medium for allowing the embryos to mature into larvae. The number of larval survivors to the total number of viable embryos produced by each breeding pair was estimated as the fecundity rate.

Fecundity Rate = (Total number of live fry/ Total number of fertile embryos) × 100.

### Euthanasia and dissection

Euthanization was performed ethically and with minimal distress to the fish by using water at 2 to 4 °C [36, 37]. The fish were dissected to remove the viscera and to expose the ovary and testis. The ovary was identified as a large-sized, lobed, cream-coloured organ with easily identifiable yolk filled eggs within. The testis was identified as a large-sized, semi-transparent, and lobed structure.

### Cytological staining with hematoxylin–eosin

The cytology smears of the ovary and testis were stained with hematoxylin and eosin for 2 min each using standard procedures. Excess stain was washed multiple times in PBS at pH 7.4 and images were captured using the image viewer software under a 40X bright-field objective (Labomed lx 400 microscope, Labomed, Los Angeles, CA, USA). Triplicate fields were randomly chosen and the cells per field were quantified using Qupath software [37].

### Ovarian reserve of eggs (Follicle count)

The ovary of an adult zebrafish is identified as a thick structure with extensive lobules. The developmental cycle of the oocyte is asynchronous containing eggs at various stages of development [38]. The fully matured egg, called Final Growth (FG), undergoes development through different stages, Stage I- Primary growth (PG), Stage II- Pre-Vitellogenic (PV), Stage III- Early vitellogenic stage (EV), Stage IV- Late Vitellogenic (LV) stage.

The ovary was dissected gently to prevent any damage to the gonad and was washed twice in PBS at pH 7.4. The oocytes at different maturation phases were gently dispersed onto a counting slide. The slide was observed under the 40X objective of a stereo-microscope (Labomed, Los Angeles, CA). The follicles were identified by the presence of yolk and the different stages were identified by their morphological features. The number of follicles at each stage of development were counted and estimated as the ovarian reserve of eggs.

### Quantification of ovarian cysts

The presence of cysts in the ovary is a sign of immature follicles that do not form fertilizable eggs and therefore, increased infertility in female zebrafish. The cysts were identified as a dark mass of cells that lack yolk and are irregularly shaped under a 40X objective of a stereo-microscope (Labomed, Los Angeles, CA).

### Study end-points for male zebrafish

Male zebrafish in the different treatment groups were treated with either SLSO or clomiphene as a reference drug. Treatments were similar to those for the female zebrafish for 14 days. On the 15^th^ day, individual fish were allowed to spawn with healthy females (1:1 ratio) to estimate the fertility rate. In addition, the sperm count and motility were also determined.

The testis was isolated and the cytological smear was stained with hematoxylin–eosin to assess the distribution of the male gametes at different developmental stages to determine the recovery from fertility impairment.

### Sperm count in male zebrafish

Fish were anaesthetized in water at 17 °C until the operculum movement came to a stop [39]. Each fish was handled separately one at a time. They were placed in a holding chamber with the ventral side up and the cloacal region was gently wiped clean with a paper towel. This ensured that any residual moisture did not activate the sperm prematurely. A capillary tube of 2 µL capacity with an attached aspirator was positioned at the cloaca and the testicular region was gently massaged with padded forceps. The resultant ejaculate was collected using the capillary tube and immediately transferred to a glass slide for microscopic counting. Images were captured and the total number of sperm was determined using Qupath software.

### Sperm motility

The sperm was freshly collected as mentioned previously and the motility was assessed according to the protocol devised by Selvaraj et al. with modifications [40,41]. Briefly, a known amount of the ejaculate was diluted to 20 µL using Hank’s Balanced Salt Solution (HBSS; 300 mOsmol/kg). The sample was centrifuged at 428 × g for 5 min and re-suspended in 1 mL of HBSS. After the numbers of sperm were counted, they were centrifuged again at 285 × g and finally re-suspended at a concentration of approximately 1 X 10^8^ sperm per µL of HBSS. If the sperm count was below the desired number, then the entire sample was re-suspended in 1 µL and used for the analysis. Following this, 1 µL of sperm was transferred to a clean glass slide and activated using 9 µL deionized water. Thereafter, the motility of the sperm was recorded using a 10X objective.

The number of moving objects were tracked using the wrMTrck plugin in the Z project [42]. The scale was set according to the video resolution and the cumulative number of moving objects was considered as the progressive motility of spermatozoa and the percentage of motility was calculated.

### Data analysis and statistics

Data are expressed as mean ± Standard Deviation (SD) of observations. Statistical analysis was performed using GraphPad Prism 7.04 (GraphPad Software Inc., San Diego, CA, USA). One-way ANOVA followed by Tukey’s multiple comparisons test was used to detect the significance of the data. In the analysis, the fertility impaired group was compared to the healthy control group, while the reference standard and SLSO treatment groups were compared to the fertility impaired group. *p*-value less than 0.5 was considered significant.

## Data Availability

The data presented in this study can be made available upon request from the scientific community at the discretion of the corresponding author. Samples of the compounds are available from the author on request for suitable academic research.

## Abbreviations

SLSO: *Shivlingi* Seed oil
ENU: N-ethyl-N-nitrosourea
GC–MS/MS: Gas Chromatography-Mass Spectrometry
HPTLC: High Performance Thin Layer Chromatography
DHA: Docosahexanoic acid
